# A New Synopsis of the Natural Order of Diseases, &c.

**Published:** 1841-07-01

**Authors:** 


### A NEW SYNOPSIS, OR THE NATURAL ORDER OF DISEASES:—WITH A NEW PATHOLOGY OF FEVER AND INFLAMMATION. By *Robert Stevens*, M.R.C.S. London: Highley, 1841.

THE successive failures of so many able writers, from Sauvages to Good inclusive, in constructing a satisfactory classification of diseases, prove that it is an undertaking of no ordinary difficulty. What practitioner attributes any portion of the success he may have acquired in the investigation or treatment of disease to aid derived from nosological systems? Nay, is he not assured that had he adhered rigidly to their artificial arrangements, he would have frequently been betrayed into many errors, impossible to an enlightened, although, by them, unaided observation? Teachers of medicine are becoming more and more persuaded of this, and hence some of the ablest professors have reverted to the topographical mode of demonstrating diseases. Objectionable as this plan may be, as involving much repetition, and neglecting general principles, it is probably, in the present state of our knowledge, the best; for, if it does not supply all the information required on commencing practice, it possesses at least the negative advantage of leaving the mind unencumbered by a mass of useless verbiage, simulating knowledge, and obstructive rather than available to future self-improvement. It is a sad thing for the young practitioner to find that he cannot treat his patients satisfactorily until he has unlearnt what he has been at much pains to acquire.

We do not say that nosologies in themselves are useless or hurtful: could a truly scientific and natural one be constructed, its utility in aiding the diagnosis, description, and treatment of disease would be unquestionable; but we fear the obstacles which oppose themselves to its construction are well-nigh insurmountable. However the successes which have attended the efforts of naturalists, may have excited the hopes and exertions of physicians, yet they have found that diseases are not to be arranged with the same ease as the objects of zoological or botanical research. The structure, history, &c. of these last are well known, their resemblances and differences easily ascertained, and hence their arrangement in systems according to their natural affinities practicable. How is this possible with diseases, whose characters are so variable, or seem to us to be so from our ignorance of their true nature, and whose affinities are so ill-known, that in every nosological system we find some such incongruities as the placing together "the itch and broken bones" in the system of Cullen, or the toothache and prolapsus ani in that of his critic Mason Good. However satisfactory it would be to be able to classify diseases according to their pathology, its impossibility is at once seen, when we consider of how few diseases the true pathology is known; and we should imagine there can be no question that it is better to practise without a nosology at all, than with one founded upon erroneous pathology. Still the attempt to improve this state of things must not be given up in despair, and we are therefore very glad to see another work appear addressed to this object, although we regret to add that it does not seem to us to have attained it.

Mr. Stevens's natural system would seem to be but a modification and extension of that of Dr. Brown, speaking of which, he thus expresses himself:—

"But Dr. Brown attempted a natural arrangement of diseases, dividing them into two classes: namely, diseases of excitement, and diseases of depression. Yet, from the great defects in the pathological knowledge of the day, and other causes, his arrangement was very imperfect; many diseases being palpably out of place. The plan, therefore, appeared to be utopian and was abandoned; but it contributed not a little to the overthrow of the Brunonian doctrines, that the principles upon which the theory itself depended, were imprudently strained. Excitability was confounded with increased vitality, whereas it is simply an undue expenditure only, an extravagant using up, as it were, of what was called the sensorial or vital power. Hence, sthenic affections, such as inflammation, are diseases of excitement only, not of increased vitality; a very important distinction, and one which most satisfactorily accounts for the subsequent depression. But the asthenic diseases are directly dependent upon diminished or defective power; and, therefore, the only defect in this system seems to be, that the sthenic and asthenic diseases are not truly and perfectly contrasted; nevertheless they are sufficiently so, to indicate in a nosological table, with the additional information conveyed by the collateral arrangements, not only much of the pathology of every disease, but also much of the principles of treatment." 4.

Following out this idea, the author considers that all diseases may be naturally arranged into the two classes of sthenic and asthenic; each class being composed of nine orders.

| Class 1.—Sthenicæ. | Class 2.—Asthenicæ. |
|---|---|
| Order 1, Febres | Order 1, Marcores |
| 2, Phlegmasiæ | 2, Adynamiæ |
| 3, Eruptiones | 3, Cachexiæ |
| 4, Impetigines | 4, Parisiticæ |
| 5, Tumores | 5, Ectopiæ |
| 6, Hæmorrhagiæ | 6, Profluviæ |
| 7, Spasmi | 7, Dyscinesiæ |

| | |
|---|---|
| 8, Dysorexiæ | 8, Dysæthesiæ |
| 9, Vesaniæ | 9, Dementiæ. |

Mr. Stevens considers that each order in the sthenic column is the natural contrast to the order placed opposite it in the asthenic column. There can be no question that diseases manifest either a sthenic or asthenic character, but we can by no means agree that many of these arranged under the above orders are *essentially* sthenic or asthenic; although they may be so in particular individuals, or in different stages of their progress. Why should hysteria, singultus or stricture, of the order spasmi, be considered essentially sthenic,—and delirium tremens, dyspepsia, jaundice, and rheumatism, essentially asthenic? If this were indeed the case, the treatment of disease, by means of a nosological table, would be a far simpler matter than we find it to be without one: but the symptoms, the treatment required, and the morbid anatomy in the various diseases, alike prevent our believing it. We think then the author's system is fanciful, and like all others that we have ever seen, it is vitiated by the grouping together of many diseases having no natural affinity, and the separation of others which should be approximated. We have only space to glance at some of the genera.

The first order *Febres* does not contain Typhus, which is removed to among the Adynamiæ. *Phlegmasiæ* contains Phthisis, and of course the various *ites;* but we do not see why Apostema (Abscess) should be elevated into the rank of a genus, when it is only one of the results of inflammation. Erythema and Erysipelas have been properly removed from among the *Eruptions* to this order, although the same observation will not apply to Lepra, which is placed among the impetigines. Of the *Impetigenes*; the author says:—" this order contains diseases depending upon specific poisons, which being once introduced into the system, continue to infect and propagate;—that is they do not run out their course in a limited time, like small-pox, leaving the system free, but, on the other hand, overcome the constitutional restorative powers, and yield to nothing but art." He enumerates three genera—Syphilis, Lepra, Elephantiasis, a number obviously too small or too great. Among the *Hæmorrhages* we find Sanguineous Apoplexy placed. The order *Spasmi* (" Abnormal muscular action: generally from the involuntary reflex action of the nerves, from irritation or injury of their peripheral extremities"), contains fifteen genera, among which are Wry-neck, Tetanus, Chorea, Hysteria, Epilepsy, Pertussis, Asthma, Colic, and Stricture. *Dysorexiæ* or depraved appetites, contains Bulimia, Polydipsia, Pica, and Salacitas: and *Vesaniæ*, Hallucinatio Iracundia and Mania.

In the second class we have as the contrast of Febres, the *Marcores*, which contains but one genus, Marasmus. The *Adynamiæ* or "Physiological contrast of the Phlegmasiæ," contains twenty-one genera, among which we find Apoplexia *Atonica*, Paralysis, Angina Pectoris, Irritabilitas Nervosa, Typhus, Cholera Asphyxia, Pestis, Dyspepsia, Jaundice, Chlorosis, Gangrene and Necrosis. We are at a loss to perceive the nature of the natural contrast between the *Cachexiæ* and Eruptiones (as indeed between several others); this order contains among other genera, Rheumatism, Gout, Calculus, Rickets, the Dropsies, Cataract, Scrofula, Cancer.

*Parisiticæ*, contains the Itch, Hydatids, Worms, &c. *Ectopiæ*, contrasted with Tumors, contains Aneurism, Varix, Piles, Nævus, Intus-susception and Hernia. *Profluviæ*, contrasted with active hæmorrhages, contains Epiphora, Gleet, Diarrhœa, Leucorrhœa, Menorrhagia, &c. *Discynesiæ* ("inefficient muscular action, being neither spasm nor paralysis ; but *mostly* a want of harmony in the action of the component muscles of an organ from nervous defect") contains Stammering, Dumbness, Aphonia, Dysphagia and Angina Pectoris. *Dysæthesiæ* contains Amaurosis, Dysopia, Impaired Smell and Taste, Deafness, Parapsis and Constipation. *Dementiæ* contains Nostalgia, Fatuitas, Amentia. To these are added an Appendix of *Symptomatic* Affections, containing Coma, Cephalalgia, Delirium, Emphysema, Hypochondriasis and Nightmare.

It will be seen, that from a nosology thus constructed, little or no assistance is to be derived by practitioner or student. This is no reflection upon its author; he has had to cope with difficulties, which, in the present state of our knowledge are not yet to be subdued, while in some respects his system manifests an improvement upon that of his predecessors. Of the pathology of *fever*, he thus speaks.

"In fever the ganglial nervous power is augmented, as well as, not only nutrition, but the counterpart absorption and redigestion or decomposition of the old material; and as there is deficient appetite, the system is thus left to prey upon itself, the excitement called fever soon merging by exhaustion into a typhoid form. So fever exists not only where the vital actions are excited by the augmented circulation of good arterial blood, but also where the system has preyed upon itself, even when prostration and debility are induced. Hence the typhoid form, and though no definition can well explain both these opposite modifications of fever, yet their separate existence as different states of the same morbid action is perfectly intelligible. For when febrile action is continued, not only is there a quicker expending of the nutritious particles of the blood, but also the counterpart absorption of old material is equally augmented, the nutritious materials being so quickly worked and expended, that shortly there is no longer the proper stimulus for the nervous centres, namely, good arterial blood, and therefore the vitality is depressed, and the patient in a state of low typhus, the circulating fluids being attenuated and the general fibre relaxed, purpura supervening and a tendency to decomposition. Whilst at the commencement of fever, when there was a quick and augmented circulation of good arterial blood going on, the patient was in a state of restless and delirious excitement. Thus, both these opposite states, result from the same morbid action, and where the strong form of fever is allowed to run very high, it soon merges by exhaustion into the low and typhoid form. But art ingeniously directed, can forestall this, and ensure the safety of the patient............

.......... "It is plain that fever manifests itself as an augmentation of the ganglial nervous supply, which governs the circulation and nutrition; also it is equally plain that the induction of faintness averts nutrition in toto. So also that medicines which produce nausea can withhold or diminish the ganglio-nervous supply, and thus afford a strong hold upon fever, in its stages of excitement; and these are the very medicines which also relieve the chief external characteristic of fever, namely, dryness of the skin, therefore fever may be cut short or reduced by artificial means, before defibrinization and exhaustion can take place." 28.

Respecting the pathology of *inflammation*, the following are some of his observations.

"It is the state of obstruction in the capillary vessels which causes the effusion of lymph; and, therefore, also the first process of inflammation. Obstruction in the course of the capillary circulation may occur in the following modes: 1st. If any abnormal deposit take place, such as tubercular matter, when it has considerably accumulated, it may press laterally on the capillary vessels, and obstruct them. If this be the case, it causes the effusion of lymph and inflammation, as will be explained presently. 2. If any portion of the texture of the body lose its vitality, it is an obstruction to the direct course of the vessels; a line of demarcation is formed, and there the effusion of lymph occurs. 3. If continued external pressure be made upon any part of the surface of the body, obstruction of the superficial vessels is the consequence." 39.

For the observations respecting the suppurative and ulcerative processes of inflammation, we have not room; nor do we perceive that they contain any novelty.

The definitions of the various diseases are concise and exact, and the principles of their treatment usually judicious. An appendix upon the relations of the medical profession with the public, proves the author to be an upright practitioner, zealous for the suppression of professional quackery and humbug: but we do not see that this is to be brought about, by such a registration of deaths as would show in which medical man's practice these preponderated, or by a more free criticism of each other's practice in unfavourable cases than is at present permitted by professional etiquette. Agreeing with the author, as to the almost hopelessness of the task of endeavouring to instruct the ignorant public in the better appreciation of who are really conscientious and able practitioners, still we think this would be the only means of improving the state of things he deplores.

